# Common mental disorders and intimate partner violence against pregnant women living with HIV in Cameroon: a cross-sectional analysis

**DOI:** 10.1186/s12884-021-03673-0

**Published:** 2021-03-04

**Authors:** Angela M. Parcesepe, Evette Cordoba, John A. Gallis, Jennifer Headley, Berenger Tchatchou, John Hembling, Claudian Soffo, Joy Noel Baumgartner

**Affiliations:** 1 Department of Maternal and Child Health, Gillings School of Global Public Health, University of North Carolina at Chapel Hill, CB# 7445, Chapel Hill, NC 27599 USA; 2 University of North Carolina at Chapel Hill, Carolina Population Center, Chapel Hill, NC USA; 3 Department of Epidemiology, Gillings School of Global Public Health, University of North Carolina at Chapel Hill, Chapel Hill, NC USA; 4 Duke Global Health Institute, Duke University, Durham, NC USA; 5 Department of Biostatistics and Bioinformatics, Duke University, Durham, NC USA; 6 Catholic Relief Services, N’Djamena, Chad; 7 Catholic Relief Services, Baltimore, MD USA; 8 Consultant, Yaounde, Cameroon

**Keywords:** Violence, Mental health, HIV, Pregnancy, Africa

## Abstract

**Background:**

Women living with HIV are at increased risk of poor mental health and intimate partner violence (IPV). Mental health disorders have been consistently associated with suboptimal HIV-related outcomes. Little is known about the prevalence or correlates of mental health disorders among pregnant women living with HIV in sub-Saharan Africa.

**Methods:**

This study assessed the prevalence of probable common mental disorders (CMD), i.e., depressive or anxiety disorders**,** and the relationship between probable CMD and recent IPV among pregnant women living with HIV in Cameroon. The sample included 230 pregnant women living with HIV aged > 18 enrolled in care at 10 HIV clinics in Cameroon. Probable CMD was assessed with the WHO Self Reporting Questionnaire (SRQ-20). Multivariable logistic regression was conducted to assess the relationship between IPV and probable CMD.

**Results:**

Almost half (42%) of participants had probable CMD using a 7/8 cut-off of the SRQ-20. Emotional, physical, and sexual IPV were reported by 44, 37, and 31% of respondents, respectively. In multivariable regression analyses, all forms of IPV assessed were significantly associated with greater odds of probable CMD.

**Conclusions:**

Pregnant women living with HIV in Cameroon had a high prevalence of probable CMD and IPV. Screening and services to address IPV and mental health are urgently needed for this population. Integrated interventions to both prevent and screen and address IPV and probable CMD should be developed, implemented, and evaluated.

## Background

Intimate partner violence (IPV) is common and associated with a cascade of negative physical and mental health outcomes for women including an increased risk of HIV acquisition [1]. IPV can take many forms including physical, sexual, and emotional IPV as well as controlling behaviors. Research has found that IPV increases risk of HIV acquisition among women by 50% [2]. IPV can lead directly to HIV acquisition through forced sex with an individual living with HIV. Risk of HIV acquisition also increases with genital injuries or lacerations, which may result from sexual IPV. IPV can also indirectly lead to HIV acquisition through increased sexual risk behaviors and limited ability to negotiate behaviors that prevent HIV. Among women living with HIV, IPV has been associated with suboptimal HIV treatment outcomes, including lower antiretroviral therapy (ART) use, low CD4 count, poor ART adherence, and lack of viral suppression [3, 4].

Women are at increased risk of experiencing IPV during pregnancy [5, 6]. IPV during pregnancy has been associated with adverse pregnancy outcomes, including preterm birth, and low birthweight [7]. Given the relationships between IPV, HIV, and pregnancy, pregnant women living with HIV may be at particularly high risk of IPV.

A growing body of evidence suggests that women living with HIV are also at increased risk of poor mental health [8–10]. Mental health disorders have been associated with suboptimal HIV-related outcomes including poor ART adherence, lack of viral suppression, faster disease progression, and AIDS-related mortality [11–13]. Likewise, perinatal (antenatal and postnatal) mental health disorders are the most common complication of pregnancy among women globally and have been associated with significant maternal morbidity [7, 14]. Among women, untreated perinatal mental health disorders have been associated with poor quality of life, reduced functioning, and postpartum mental health disorders [14]. Untreated perinatal mental health disorders have also been associated with adverse infant health outcomes including preterm birth, low birth weight, intrauterine growth restriction, and behavioral difficulties [7, 14, 15].

Women living with HIV constitute half of all people living with HIV (PLHIV) globally, 58% of all PLHIV in sub-Saharan Africa, and 65% of PLHIV in Cameroon [16, 17]. Advancements in ART have made the elimination of mother-to-child-transmission of HIV feasible and a global public health priority. In recognition of this, the World Health Organization (WHO) endorsed a global health strategy that involves working towards a goal of no new HIV infections in infants by 2020 [18]. In addition, the Joint United Nations Programme on HIV/AIDS (UNAIDS) and the United States President’s Emergency Plan for AIDS Relief (PEPFAR) launched the *Start Free Stay Free AIDS Free* framework for ending AIDS in children, adolescents and young women by 2020 [19]. Cameroon, one of 23 focus countries for the *Start Free Stay Free AIDS Free* framework, accounts for approximately 3% of children who acquire HIV through vertical transmission globally [19]. Maternal-to-child-transmission of HIV in Cameroon is largely driven by low rates of ART initiation during pregnancy and poor retention in HIV care during pregnancy [19]. Only 80% of pregnant women living with HIV in Cameroon received ART for prevention of mother-to-child transmission (PMTCT) of HIV compared to more than 90% throughout Eastern and Southern Africa [19]. In addition, 20% of pregnant women who initiate ART in sub-Saharan Africa disengage from care before giving birth [19].

Despite the consistent relationship between IPV, poor mental health, and HIV, little is known about the prevalence of IPV or mental health disorders among pregnant women living with HIV in sub-Saharan Africa, the region with the greatest burden of the HIV epidemic globally. A systematic review of IPV against women living with HIV in sub-Saharan Africa identified just 12 quantitative studies for inclusion, five of which were focused on pregnant women living with HIV [20]. In addition, factors associated with mental health disorders during the antenatal period among African women living with HIV remain poorly understood. Greater understanding of the burden and relationship between IPV and mental health symptoms among pregnant women living with HIV can inform the development of interventions to prevent and address IPV and perinatal mental health disorders among this population and has the potential to improve HIV outcomes among pregnant women living with HIV and reduce vertical transmission of HIV. The objectives of this paper are 1) to estimate the prevalence of recent IPV and probable common mental disorders (CMD) among pregnant women living with HIV in Cameroon, 2) to examine the relationship between CMD and four forms of IPV (physical, sexual, and emotional IPV, and controlling behavior), and 3) to identify additional factors associated with CMD among this population.

## Methods

Data were collected as part of an impact evaluation of an early childhood development intervention implemented by Catholic Relief Services and conducted in 10 PMTCT health clinics in the Djoungolo or Nkoldongo Health Districts of Yaoundé, Cameroon. Individuals were eligible to participate in the study if they were 18 years of age or older, living with HIV, in their third trimester of pregnancy, and residing in the study districts. The current analysis used baseline data from the 230 pregnant women living with HIV who enrolled in the parent study. Data collection consisted of a structured interview during the study participants’ third trimester of pregnancy that included questions on mental health, IPV, and socio-demographic background.

### Measures

#### Mental health

Mental health was measured with the WHO Self-Reporting Questionnaire (SRQ-20). The SRQ-20 is a mental health screening tool which includes questions related to depression, anxiety, and somatic symptoms in the last 30 days [21]. Scores range from 0 to 20 and a cutoff score determines probable CMD. The SRQ-20 was developed to screen for probable CMD (including depressive, anxiety and somatic symptoms), particularly in low-and middle-income countries (LMIC) [21]. The SRQ-20 has been used extensively to screen for probable CMD in primary care settings throughout sub-Saharan Africa [22–24]. The SRQ-20 has also been previously used with pregnant women, including across African countries [25–27].

#### Intimate partner violence

Assessment of IPV was based on the Demographic and Health Survey (DHS) violence module and consisted of questions across four domains of IPV: controlling behavior, and emotional, sexual, and physical IPV [28–30]. Assessment of partner controlling behaviors consisted of five questions about whether the study participant’s spouse or partner: *was jealous or angry if you talked to other men; frequently accuses you of being unfaithful; does not permit you to meet your girlfriends; tries to limit your contact with family; or insists on knowing where you are at all times.* Individuals who responded no to all of these questions or yes to one of these questions were categorized as having experienced no or low levels of controlling behaviors. Individuals who responded yes to between two and four of these questions were categorized as having experienced moderate levels of controlling behaviors. Individuals who responded yes to five or six of these questions were categorized as having experienced high levels of controlling behaviors.

Assessment of emotional IPV consisted of three questions about whether the study participant’s spouse or partner in the last 12 months: *said or did something to humiliate you in front of others; threatened you or someone close to you with harm; or insulted or belittled you*. Participants who responded yes to one or more of these questions were categorized as having experienced emotional IPV.

Assessment of sexual IPV consisted of two questions about where the participant’s spouse or partner in the last 12 months: *physically forced you to have sexual intercourse with him even when you did not want to or forced you to perform other sexual acts you did not want to*. Participants who responded yes to either of these questions were categorized as having experienced sexual IPV.

Assessment of physical IPV consisted of eight questions about whether the participant’s spouse or partner in the last 12 months: *pushed you, shaken you, or thrown something at you; slapped you; twisted your arm or pulled your hair; punched you with his fist or with something that could hurt you; kicked or dragged you; tried to strangle or burn you; threatened you with a knife, gun, or other type of weapon; or attacked you with a knife, gun, or other type of weapon*. Participants who responded yes to one or more of these questions were categorized as having experienced physical IPV.

#### Socio-demographic variables

Participant socio-demographic data included participant’s age, education, relationship status, general health, asset-based socioeconomic status (SES), household hunger, employment status, and bodily pain. Household hunger in the past 4 weeks was assessed with the Household Hunger Scale [31]. This scale consisted of three questions about household hunger in the past 30 days: *was there ever no food to eat of any kind in your house because of lack of resources to get food; did you or any household member go to sleep at night hungry because there was not enough food; did you or any household member go a whole day and night without eating anything because there was not enough food*. If participants responded affirmatively to any question, they were asked about frequency of the occurrence. This instrument was developed and validated for cross-cultural use to measure a household’s ability to access food within the last 30 days. Responses to these questions were recoded, summed, and categorized as little to no hunger, moderate hunger, or severe hunger [32].

Asset-based SES was constructed by asking participants about ownership of the following six assets: electricity, radio, television, landline telephone, computer, and refrigerator. Participants were also asked about the type of fuel used for cooking (dichotomized into natural gas vs. other types) and water source (dichotomized into piped water vs. other sources). Polychoric correlation principal components analysis was used to construct asset-based SES quintiles [33–35]. Asset-based data are viewed as valid and reliable, particularly in low-resource settings, where income and expenditure-related data are often less reliable [36]. In simulations, this approach has been shown to perform better than the traditional principal components approach, which uses correlations based on multivariate normality of the assets [37]. Information about relationship status was categorized as whether or not the participant was currently cohabitating with their romantic partner. Participants were also asked how many times they had become pregnant, including their current pregnancy. Information about general health was assessed using a five-point question asking participants to rate their health on a scale including poor, fair, good, very good, and excellent. Information about bodily pain was assessed using a six-point question asking participants to rate their bodily pain in the past 4 weeks from none to very severe. Research suggests that bodily pain and mental health disorders commonly co-occur, and individuals who endorse pain are at increased risk of mental health disorders [38]. In addition, endorsement of somatic symptoms such as pain, have been shown to be elevated among survivors of interpersonal trauma [39].

### Analysis

The current analysis used baseline data from the 230 pregnant women living with HIV enrolled in the parent study. Univariate analyses were conducted to estimate the prevalence of mental health symptoms, probable CMD, and IPV. To define probable CMD, we used two distinct SRQ-20 cutoff scores. When the SRQ-20 was developed, the WHO indicated that there was no global, generally applicable cut-off for this instrument [21]. Rather, the WHO recommended that each study determine its own cut off. In recognition of the lack of a single universal cut-off and because, to the authors’ knowledge, the SRQ-20 has not been formally validated in Cameroon, we conducted our analyses using two different cut-offs - a 7/8 cutoff reflecting a more commonly used global cutoff and a 9/10 cutoff to capture only those with more severe mental health symptoms and a pressing need for mental health care [40]. The Cronbach’s alpha for the SRQ-20 was 0.78.

Bivariate analyses between SRQ-20 score cutoffs and respondent characteristics used Pearson’s chi-squared tests or Fisher’s exact tests, as appropriate. Due to the substantial portion of the sample population that reported recent suicidal ideation, we also conducted exploratory bivariate analyses between recent suicidal ideation and IPV using Pearson’s chi-squared tests. We used logistic generalized estimating equations (GEE) models with exchangeable correlation matrix (to take into account potential clustering of outcomes by clinic) to examine the relationship between IPV and probable CMD defined by each of the SRQ-20 cutoffs. Because IPV variables were correlated with each other (Pearson correlation coefficient of physical IPV and emotional IPV = 0.35; Pearson correlation coefficient of physical IPV and sexual IPV = 0.32), multivariable regression models were run separately to examine the relationship between each type of IPV (i.e., controlling behaviors, and emotional, physical, and sexual IPV) and probable CMD while adjusting for covariates. In addition, multivariable regression models were also run using a multi-category IPV variable categorized as: no experiences of emotional, sexual, or physical IPV, emotional IPV only, and physical or sexual IPV (with or without emotional IPV). This multi-category variable allows us to understand the unique relationship between emotional IPV and probable CMD [41]**.** Multivariable models adjusted a priori for participant’s age. A *p*-value of < 0.05 in bivariate analyses guided interpretation of statistical significance and additional model variable selection. Education was correlated with household hunger and was not included in multivariable models. General health status was correlated with severity of bodily pain and was not included in multivariable models. Ethical approvals were obtained by Duke University Campus IRB and the Comite National d’Ethique de la Recherche pour la Sante Humaine of Cameroon.

## Results

Among the 230 women surveyed, one-quarter (26.7%) of participants had probable CMD using the 9/10 cut-off of the SRQ-20 while 42.2% screened positive for CMD using the less conservative 7/8 cut-off score. In addition, a substantial minority (14.8%) endorsed recent suicidal ideation (Table 1). The mean (SD) SRQ-20 score was 6.8 (4.0).

**Table 1:** SRQ-20 results for probable common mental disorder among pregnant women living with HIV in Yaoundé, Cameroon

SRQ-20 Questions	Total Sample *N* = 230 No. responding yes (%)
Do you often have headaches?	92 (40.0)
Is your appetite poor?	95 (41.3)
Do you sleep badly?	105 (45.6)
Are you easily frightened?	123 (53.5)
Do your hands shake?	29 (12.6)
Do you feel nervous, tense, or worried?	154 (67.0)
Is your digestion poor?	72 (31.3)
Do you have trouble thinking clearly?	39 (17.0)
Do you feel unhappy?	91 (39.6)
Do you cry more than usual?	140 (60.9)
Do you find it difficult to enjoy your daily activities?	68 (29.6)
Do you find it difficult to make a decision?	93 (40.4)
Is your daily work suffering?	62 (27.0)
Are you unable to play a useful part in life?	43 (18.7)
Have you lost interest in things?	57 (24.8)
Do you feel that you are a worthless person?	44 (19.1)
Has the thought of ending your life been on your mind?	34 (14.8)
Do you feel tired all the time?	72 (31.3)
Do you have uncomfortable feelings in your stomach?	69 (30.0)
Are you easily tired?	81 (35.2)

Most (78.7%) respondents were 25 years of age or older, had begun but did not complete secondary education (56.5%), and were living with a romantic partner (69.1%) (Table 2). Approximately one-fifth (19.6%) of respondents endorsed moderate or severe household hunger. Half (50.2%) of respondents had been employed in the past 3 months. Approximately one-third (29.7%) of participants reported fair or poor health and one-quarter (27.4%) endorsed moderate or severe bodily pain in the past 4 weeks.

**Table 2:** Sociodemographic characteristics and probable CMD among pregnant women living with HIV in Cameroon by SRQ-20 cutoff score

	Total Sample ***N*** = 230 N (%)	Probable common mental disorder SRQ-20 7/8 cut-off			Probable common mental disorder SRQ-20 9/10 cut-off		
		0–7 score N (%)	8+ score N (%)	***p***-value	0–9 score N (%)	10+ score N (%)	***p***-value
**Total sample**		133 (57.8)	97 (42.2)		171 (74.3)	59 (25.7)	
**Age**				0.83			0.87
18–24	49 (21.3)	29 (21.8)	20 (20.6)		36 (21.0)	13 (22.0)	
25+	181 (78.7)	104 (78.2)	77 (79.4)		135 (79.0)	46 (78.0)	
**Education**				0.17			0.02
None to primary	45 (19.6)	22 (16.5)	23 (23.7)		26 (15.2)	19 (32.2)	
Some secondary	130 (56.5)	74 (55.6)	56 (57.7)		102 (59.6)	28 (47.5)	
Completed secondary or more	55 (23.9)	37 (27.8)	18 (18.6)		43 (25.5)	12 (20.3)	
**Relationship status**				0.04			0.56
Not living with romantic partner	71 (30.9)	34 (25.6)	37 (38.1)		51 (29.8)	20 (33.9)	
Living with romantic partner	159 (69.1)	99 (74.4)	60 (61.9)		120 (70.2)	39 (66.1)	
**Number of pregnancies (including current)**				0.12			0.61
One pregnancy	21 (9.1)	13 (9.8)	8 (8.2)		14 (8.2)	7 (11.9)	
2–3 pregnancies	99 (43.0)	64 (48.1)	35 (36.1)		76 (44.4)	23 (39.0)	
4 or more pregnancies	110 (47.8)	56 (42.1)	54 (55.7)		81 (47.4)	29 (49.2)	
**General health status**				<.0001			0.0002
Fair/Poor	68 (29.7)	25 (18.8)	43 (44.8)		41 (24.0)	27 (46.5)	
Good	100 (43.7)	60 (45.1)	40 (41.7)		74 (43.3)	26 (44.8)	
Very Good/ Excellent	61 (26.6)	48 (36.1)	13 (13.5)		56 (32.7)	5 (8.6)	
Missing	1						
**Asset-based SES by quintiles**				0.25			0.94
Lowest Quintile	40 (17.4)	21 (15.8)	19 (19.6)		29 (16.7)	11 (18.6)	
Lower Middle Quintile	43 (18.7)	21 (15.8)	22 (22.7)		33 (19.3)	10 (16.9)	
Middle Quintile	54 (23.5)	38 (28.6)	16 (16.5)		42 (24.6)	12 (20.3)	
Upper Middle Quintile	40 (17.4)	23 (17.3)	17 (17.5)		29 (17.0)	11 (18.6)	
Upper Quintile	53 (23.0)	30 (22.6)	23 (23.7)		38 (22.2)	15 (25.4)	
**Household hunger**				0.0002			0.006
None/low	185 (80.4)	119 (89.5)	66 (68.0)		146 (85.4)	39 (66.1)	
Moderate	36 (15.6)	10 (7.5)	26 (26.8)		20 (11.7)	16 (27.1)	
Severe	9 (3.9)	4 (3.0)	5 (5.2)		5 (3.0)	4 (6.8)	
**Employment in last 3 months**				0.54			0.97
No	113 (49.8)	68 (51.5)	45 (47.4)		84 (49.7)	29 (50.0)	
Yes	114 (50.2)	64 (48.5)	50 (52.6)		85 (50.3)	29 (50.0)	
Missing	3						
**Bodily pain (past 4 weeks)**				0.05			0.19
None/ Very mild/ Mild	167 (72.6)	103 (77.4)	64 (66.0)		128 (74.8)	39 (66.1)	
Moderate/ Severe	63 (27.4)	30 (22.6)	33 (34.0)		43 (25.2)	20 (33.9)	
**District**							
Nkoldongo	146 (64.0)	83 (63.4)	63 (65.0)	0.80	107 (63.3)	39 (66.1)	0.70
Djoungolo	82 (36.0)	48 (36.6)	34 (35.1)		62 (36.7)	20 (33.9)	
Missing	2						

At both cut-offs, a greater proportion of individuals with probable CMD reported fair or poor health compared to individuals with no probable CMD. Similarly, at both cut-offs, a greater proportion of individuals with probable CMD reported moderate or severe household hunger compared to individuals with no probable CMD.

All forms of IPV were commonly reported (Table 3). Most participants (57.3%) reported having experienced moderate controlling behaviors and 44.5% reported emotional IPV in the last 12 months (Table 4). Approximately one-third reported physical IPV (36.6%) or sexual IPV (31.3%) in the past 12 months. When taken together, most participants (62.6%) reported experiencing at least one form of emotional, physical, or sexual IPV and 14.3% reporting experiencing all three forms of IPV (data not shown). At both cut-offs, a greater proportion of individuals with probable CMD reported having experienced emotional, physical, and sexual IPV. At the 9/10 cut off only, a greater proportion of individuals with probable CMD reported having experienced moderate or high levels of controlling behaviors. A greater proportion of individuals reporting recent suicidal ideation reported having experienced recent physical IPV and recent emotional IPV.

**Table 3:** Recent Intimate Partner Violence against Pregnant Women Living with HIV in Yaoundé, Cameroon

Variable	Total Sample *N* = 227 No. responding yes (%)
**Controlling behavior**	
Jealous or angry if you talk to other men	43 (18.9)
Accuses you of being unfaithful	165 (72.7)
Does not permit you to meet your girl friends	166 (73.1)
Tries to limit your contact with your family	185 (81.5)
Insist on knowing where you are at all times	91 (40.1)
**Emotional IPV**	
Said or done something to humiliate you in front of others	173 (76.2)
Threatened you or someone close to you with harm	186 (81.9)
Insulted or belittled you	148 (65.2)
**Physical IPV**	
Pushed you, shaken you, or thrown something at you	55 (24.2)
Slapped you	66 (29.1)
Twisted your arm or pulled your hair	42 (18.5)
Punched you with his fist or with something that could hurt you	28 (12.3)
Kicked you or dragged you	27 (11.9)
Tried to strangle you or burn you	15 (6.6)
Threatened you with a knife, gun, or other type of weapon	5 (2.2)
Attacked you with a knife, gun, or other type of weapon	5 (2.2)
**Sexual IPV**	
Forced you to have sexual intercourse	64 (28.2)
Forced you to perform other sexual acts	32 (14.1)

**Table 4:** IPV, probable CMD, and suicidal ideation among pregnant women living with HIV in Cameroon by SRQ-20 score

Variable	Total Sample (***N*** = 230) N (%)	Probable common mental disorder SRQ-20 7/8 cutoff			Probable common mental disorder SRQ-20 9/10 cutoff			Recent suicidal ideation		
		0–7 score ***N*** = 133 N (%)	8+ score ***N*** = 97 N (%)	***p***-value	0–9 score ***N*** = 171 N (%)	10+ score ***N*** = 59 N (%)	***p***-value	No ***N*** = 196 N (%)	Yes ***N*** = 34 N (%)	***p***-value
**Controlling behaviors**				0.06			0.01			0.25
No/low controlling behaviors	78 (34.4)	53 (39.9)	25 (26.6)		66 (38.6)	12 (21.4)		69 (35.4)	9 (28.1)	
Moderate controlling behaviors	130 (57.3)	72 (54.1)	58 (61.7)		95 (55.6)	35 (62.5)		112 (57.4)	18 (56.3)	
High controlling behaviors	19 (8.4)	8 (6.0)	11 (11.7)		10 (5.9)	9 (16.1)		14 (7.2)	5 (15.6)	
Missing	3									
**Emotional IPV**				0.0001			< 0.0001			0.03
No	126 (55.5)	88 (66.2)	38 (40.4)		108 (63.2)	18 (32.1)		114 (58.5)	12 (37.5)	
Yes	101 (44.5)	45 (33.8)	56 (59.6)		63 (36.8)	38 (67.9)		81 (41.5)	20 (62.5)	
Missing	3									
**Physical IPV**				0.001			0.02			0.01
No	144 (63.4)	96 (72.2)	48 (51.1)		116 (67.8)	28 (50.0)		130 (66.7)	14 (43.8)	
Yes	83 (36.6)	37 (27.8)	46 (48.9)		55 (32.2)	28 (50.0)		65 (33.3)	18 (56.3)	
Missing	3									
**Sexual IPV**				0.005			0.0001			0.10
No	156 (68.7)	101 (75.9)	55 (58.5)		129 (75.4)	27 (48.2)		138 (70.8)	18 (56.3)	
Yes	71 (31.3)	32 (24.1)	39 (41.5)		42 (24.6)	29 (51.8)		57 (29.2)	14 (43.8)	
Missing	3									

In multivariable analyses, when each type of violence was assessed separately, emotional, physical, and sexual IPV were associated with significantly greater odds of probable CMD at both the 7/8 and 9/10 cut-offs (Table 5). More specifically, participants who reported having experienced recent emotional IPV had approximately three times the odds of probable CMD (7/8 cutoff: aOR 2.9 [95% CI 1.9, 4.5]; 9/10 cutoff: aOR 3.5 [95% CI 2.0, 6.0]) compared to those who did not report recent emotional IPV. Those who reported having experienced recent physical IPV had approximately two times the odds of probable CMD (7/8 cutoff: aOR 2.6 [95% CI 1.5, 4.7]; 9/10 cutoff: aOR 2.2 [95% CI 1.1, 4.3]) compared to those who did not experience recent physical IPV. Those who experienced recent sexual IPV had approximately two times the odds (aOR 2.1 [95% CI 1.3, 3.3]) of probable CMD at the 7/8 cutoff and almost three times the odds (aOR 2.8 [95% CI 1.6, 4.9] of probable CMD at the 9/10 cutoff. Having experienced moderate or high levels of controlling behaviors was associated with probable CMD at the 9/10 cutoff only (7/8 cutoff: aOR 1.5 [95% CI 0.9, 2.5]; 9/10 cutoff: aOR 1.8 [95% CI 1.2, 2.7]).

**Table 5:** Bivariate and multivariable associations between IPV and probable CMD among pregnant women living with HIV in Cameroon by SRQ-20 score

	Probable common mental disorder SRQ-20 7/8 cut-off					Probable common mental disorder SRQ-20 9/10 cut-off				
	Bivariate OR (95% CI)	Multivariable^**a**^ aOR (95% CI)	Multivariable^**b**^ aOR (95% CI)	Multivariable^**c**^ aOR (95% CI)	Multivariable^**d**^ aOR (95% CI)	Bivariate OR (95% CI)	Multivariable^**a**^ aOR (95% CI)	Multivariable^**b**^ aOR (95% CI)	Multivariable^**c**^ aOR (95% CI)	Multivariable^**d**^ aOR (95% CI)
**Controlling behaviors**										
None/low	1.00	1.00				1.00	1.00			
Moderate/high	1.81 (1.06, 3.08)	1.48 (0.88, 2.47)				2.60 (1.47, 4.60)	1.77 (1.17, 2.68)			
**Emotional IPV**	2.86 (1.88, 4.34)		2.92 (1.91, 4.47)			3.62 (2.10, 6.23)		3.48 (2.01, 6.04)		
**Physical IPV**	2.49 (1.50, 4.12)			2.64 (1.47, 4.73)		2.21 (1.25, 3.88)			2.20 (1.14, 4.26)	
**Sexual IPV**	2.19 (1.38, 3.48)				2.07 (1.32, 3.25)	3.02 (1.63, 5.62)				2.83 (1.64, 4.87)
**Age**										
18–24		0.99 (0.52, 1.87)	0.94 (0.47, 1.85)	1.09 (0.62, 1.90)	0.95 (0.53, 1.70)		1.20 (0.77, 1.88)	1.18 (0.68, 2.03)	1.38 (0.87, 2.18)	1.09 (0.59, 2.03)
25+		1.00	1.00	1.00	1.00		1.00	1.00	1.00	1.00
**Household hunger**										
None/low		1.00	1.00	1.00	1.00		1.00	1.00	1.00	1.00
Moderate/severe		3.65 (1.63, 8.18)	3.71 (1.46, 9.41)	3.64 (1.64, 8.10)	3.38 (1.42, 8.03)		2.45 (1.37, 4.39)	2.47 (1.17, 5.22)	2.31 (1.19, 4.48)	2.36 (1.25, 4.49)
**Living with partner**										
Yes		1.00	1.00	1.00	1.00		1.00	1.00	1.00	1.00
No		1.78 (1.11, 2.86)	2.01 (1.24, 3.24)	2.09 (1.21, 3.59)	1.88 (1.12, 3.16)		1.05 (0.60, 1.81)	1.15 (0.70, 1.89)	1.15 (0.62, 2.12)	1.14 (0.62, 2.11)
**Bodily pain**										
None/Mild		1.00	1.00	1.00	1.00		1.00	1.00	1.00	1.00
Moderate/severe		1.72 (0.96, 3.08)	1.65 (0.97, 2.81)	1.84 (0.94, 3.61)	1.75 (0.99, 3.08)		1.48 (0.74, 2.98)	1.34 (0.68, 2.67)	1.53 (0.77, 3.02)	1.47 (0.71, 3.04)

^a^Model includes controlling behaviors, but not emotional, physical, or sexual IPV

^b^Model includes emotional IPV, but not controlling behaviors or physical or sexual IPV

^c^Model includes physical IPV, but not controlling behaviors or emotional or sexual IPV

^d^Model includes sexual IPV, but not controlling behaviors or emotional or physical IPV

When IPV was examined as a multi-category variable (Table 6), participants who reported having experienced only emotional IPV had three times the odds of probable CMD at the 7/8 cutoff (aOR 3.0 [95% CI 1.5, 6.1]) and over four times the odds of probable CMD [aOR 4.6 95% CI 1.6, 13.2]) at the 9/10 cutoff compared to those who did not report any IPV. Those who reported having experienced recent physical or sexual IPV (with or without emotional IPV) had over three times the odds of probable CMD at the 7/8 cutoff (aOR 3.6 [95% CI 2.0, 6.5] and approximately four times the odds of probable CMD at the 9/10 cutoff (aOR 4.3 [95% CI 1.8, 10.1]) compared to those who did not experience any recent IPV.

**Table 6:** Bivariate and multivariable associations between IPV and probable CMD among pregnant women living with HIV in Cameroon by SRQ-20 score using a multi-category IPV variable

	Probable common mental disorder SRQ-20 7/8 cut-off		Probable common mental disorder SRQ-20 9/10 cut-off	
	Bivariate OR (95% CI)	Multivariable aOR (95% CI)	Bivariate OR (95% CI)	Multivariable aOR (95% CI)
**No IPV**	1.00	1.00	1.00	1.00
**Emotional IPV only**	3.11 (1.61, 6.01)	3.00 (1.47, 6.11)	4.80 (1.81, 12.73)	4.59 (1.60, 13.18)
**Sexual or physical IPV (with or without emotional IPV)**	3.75 (2.16, 6.50)	3.62 (2.01, 6.52)	4.55 (1.84, 11.27)	4.26 (1.79, 10.11)
**Age**				
18–24		1.03 (0.56, 1.91)		1.25 (0.75, 2.09)
25+		1.00		1.00
**Household hunger**				
None/low		1.00		1.00
Moderate/severe		3.45 (1.48, 8.03)		2.45 (1.26, 4.78)
**Living with partner**				
Yes		1.00		1.00
No		1.99 (1.20, 3.31)		1.09 (0.64, 1.88)
**Bodily pain**				
None/Mild		1.00		1.00
Moderate/severe		1.54 (0.92, 2.58)		1.26 (0.64, 2.48)

Recent household hunger was significantly associated with probable CMD in all multivariable models at both the 9/10 and 7/8 cutoffs. Those who reported recent household hunger had between two and three times the odds of probable CMD compared to those who reported no or low household hunger across all multivariable models. Not living with a romantic partner was associated with significantly greater odds of probable CMD in multivariable models at the 7/8 cut-off only.

## Discussion

All forms of IPV were commonly reported among study participants. Controlling behaviors were most commonly reported with 66% of participants having experienced moderate or high levels of controlling behaviors and 89% of participants having experienced any controlling behaviors. Research with the general population of Cameroonian women that found that 82% of Cameroonian women surveyed reported controlling behaviors [42]. Approximately one-third (37%) of participants reported having experienced physical IPV in the past 12 months. The prevalence of physical IPV in the current study is higher than previous estimates of recent physical IPV among the general population of Cameroonian women (29%) and among women living with HIV in Cameroon (22%) [43, 44]. Estimates of emotional and sexual IPV were also higher in the current study as compared to estimates from the general population of Cameroonian women and Cameroonian women living with HIV. Almost half (44%) of women surveyed reported having experienced emotional IPV in the past 12 months. Previous research found that approximately one-third (33%) of Cameroonian women in the general population and 29% of Cameroonian women living with HIV reported recent emotional IPV [43, 44]. Almost one-third (31%) of participants in the current study reported sexual IPV in the past 12 months. Previous research has estimated that 11% of Cameroonian women in the general population and 18% of Cameroonian women living with HIV reported recent sexual IPV [43, 44]. The authors know of no previous estimates of IPV among pregnant women living with HIV in Cameroon. It is unclear why estimates of recent IPV were higher in the current study compared to previous studies. It possible that thorough interviewer training and a more targeted focus on violence in the current study led to greater disclosure of IPV compared to previous studies. On the other hand, it is possible that pregnant women living with HIV in Cameroon are at increased risk of IPV compared to other populations of women. It has been well-documented that women are at increased risk of experiencing IPV during pregnancy [5, 6]. Further, women living with HIV are at greater risk of experiencing IPV compared to the general population [45]. Thus, pregnant women living with HIV in Cameroon may be particularly vulnerable to experiences of IPV. Additional research into the prevalence of IPV among pregnant women living with HIV in Cameroon and other low-resource settings is warranted.

Emotional, physical, and sexual IPV were all significantly associated with probable CMD at both the 7/8 and 9/10 cutoffs. These findings are consistent with a systematic review of IPV and perinatal mental health disorders among women in LMICs that found that those who had experienced IPV had significantly greater odds of antenatal depression compared to those who had not experienced IPV [46]. Importantly, experiencing emotional IPV alone was significantly associated with greater odds of probable CMD. Greater attention to the prevalence and impact of emotional IPV, with or without accompanying physical or sexual IPV, is warranted. Experiencing moderate or high levels of controlling behavior was associated with probable CMD at the 9/10 cutoff only. Future research into mediators and moderators of the relationship between IPV and probable CMD, such as coping strategies, social support, and material hardship, should be explored.

IPV is a major public health problem affecting pregnant women living with HIV in Cameroon and is associated with worse mental health among this population. Our findings suggest that IPV may be higher among pregnant women living with HIV compared to both the general population of Cameroonian women and non-pregnant Cameroonian women living with HIV. IPV screening and counseling should be integrated into PMTCT services as has been recommended by the WHO [47]. Little evidence exists regarding the effectiveness of evidence-based interventions to address IPV among pregnant women living with HIV or interventions focused on both IPV and HIV-related outcomes. A systematic review of the efficacy and effectiveness of IPV interventions generally found that cognitive- and behaviorally-based interventions were associated with improved mental health [48]. A systematic review of cognitive behavioral therapy and advocacy interventions for female survivors of IPV found that both types of interventions were associated with reductions in physical and psychological, but not sexual IPV [49]. Strategies to address IPV in the context of pregnancy for women living with HIV are urgently needed. The impact of such strategies on IPV, mental health, and HIV treatment outcomes of pregnant women living with HIV should be evaluated. In addition, interventions at the community level to address social norms related to IPV should be considered as such interventions have been effective at changing social norms. Evidence of the effectiveness of such interventions to reduce the incidence of IPV remains limited [50]. Research focused on interventions to prevent or respond to IPV should adhere to ethical and safety recommendations for conducting such research that have been developed by the WHO [51].

Probable CMD was commonly endorsed among study participants, with one-quarter of participants screening positive for probable CMD at 9/10 cutoff and 42% screening positive at the 7/8 cutoff. The authors are not aware of previous estimates of probable CMD among pregnant women living with HIV in Cameroon. Study findings are similar to estimates from a systematic review of perinatal depression among African women living with HIV which found a weighted mean prevalence of antenatal depression of 23.4% and suspected antenatal depression of 45.5% [7]. It should be noted that the SRQ-20 assesses a range of mental health symptoms, including but not limited to depressive symptoms. A study of pregnant women living with HIV in Zambia which used the SRQ-20 estimated the prevalence of antenatal probable CMD to be 23% using a 6/7 cutoff, a different cutoff that was used in the current analysis, making comparisons difficult [52]. Caution is warranted when comparing findings across studies due to differences in measurement, samples, and timing of mental health assessment during pregnancy. Despite such limitations, grounding our findings in previous literature is relevant as it provides a useful context in which to interpret current study findings. Suicidal ideation was also common among this population, with 15% reporting recent suicidal thoughts. In bivariate analyses, a greater proportion of individuals reporting suicidal ideation reported recent physical IPV and emotional IPV. The data reveal that pregnant women living with HIV in Cameroon are facing a multitude of hardships and risk factors for mental health disorders. Findings highlight a critical need for screening and services to address IPV and mental health disorders for pregnant women living with HIV in Cameroon.

Integration of mental health screening and treatment into HIV care services remains limited in Cameroon and throughout low-resource settings [53]. Routine mental health screening and referral to treatment should be integrated into PMTCT services as integration of mental health and HIV care has been identified as a promising strategy to improve both mental health and HIV treatment outcomes among PLHIV [54]. While mental health services may not address all of the complex psychosocial and environmental issues that these women face, mental health care and treatment is an essential part of quality healthcare for HIV-affected populations. A study with primary care providers in Cameroon found that only 2% of providers used a standardized tool to screen for depression, despite almost all (93%) providers indicating an awareness that depression warrants medical intervention [55]. Common barriers to the integration of mental health screening and care into primary care services, such as lack of mental health training and mental health-related stigma, should be investigated for their relevancy in the context of PMTCT services. For example, a study with primary care providers in Cameroon found that less than half (49%) had received any formal mental health training [55]. In addition, two-thirds of primary care providers surveyed in Cameroon indicated that they felt uncomfortable working with patients with depression [55]. Similar to many resource-constrained countries, Cameroon faces a substantial shortage of mental health specialists. Task-shifting or task-sharing strategies in which non-specialists deliver mental health screening and interventions can increase access to evidence-based mental health care and is in line with WHO’s Mental Health Gap Action Programme (mhGAP) Intervention Guidelines [56]. Such strategies should be implemented and evaluated in PMTCT and other maternal health care settings in Cameroon.

Few evidence-based mental health interventions have been studied with pregnant women living with HIV in sub-Saharan Africa. A quasi-experimental study of an intervention which involved a cognitive-behavioral group component and individual support was associated with reduced depression symptoms compared to standard care among pregnant women living with HIV in South Africa [57]. An intervention which included weekly telephone sessions of emotional and educational support delivered by a nurse was associated with decreased depression symptoms compared to a control condition among pregnant women living with HIV in Thailand [58]. Future research should examine the effectiveness of evidence-based mental health interventions for pregnant women living with HIV in Cameroon and other low-resource settings. Such research should investigate the impact of such interventions on mental health symptoms and disorders and outcomes throughout the HIV care cascade.

Few integrated interventions focused on both IPV and CMD have been developed, implemented, and evaluated [59, 60]. This represents a critical programmatic gap that requires urgent attention, particularly as IPV and CMD commonly co-occur and have been shown to have a cyclical, bidirectional relationship. A systematic review of mental health interventions conducted in LMICs that measured both mental health and IPV outcomes identified just three interventions with integrated mental health and IPV components, all of which targeted alcohol or drug use, rather than CMD [59]. Integrated IPV and mental health interventions may be particularly suited for secondary or tertiary IPV prevention programming and for women experiencing IPV who want to maintain their partnership. Research into the effectiveness of integrated IPV and mental health interventions and the comparative effectiveness of such interventions to single-component interventions is needed.

Household hunger was common and significantly associated with probable CMD. This is consistent with previous research that found that food insecurity was associated with perinatal depressive symptoms among low-income South African women and with pregnant women in Ethiopia [61, 62]. Research from high-income countries suggests that the relationship between household hunger and mental health may be bidirectional [63]. That is, household hunger may lead to anxiety and worse mental health. Alternately, mental health symptoms may serve as a barrier to employment or social engagement, resulting in limited financial or social resources, contributing to household hunger. Indeed, approximately half of study participants reported recent unemployment. Evidence-based interventions to address household hunger and mental health simultaneously among PLHIV remain limited. However, findings suggest that interventions that focus on improving food security among pregnant women living with HIV may have mental health benefits. Interventions to improve food access should be integrated into PMTCT services. The impact of such interventions on household hunger, mental health and engagement in PMTCT services should be investigated.

Not living with a romantic partner was associated with probable CMD at the 7/8 threshold. Women not living with romantic partners may face greater household or financial responsibilities or greater material hardship compared to women living with partners. Material hardship has been associated with CMD among mothers [64]. Individuals not living with a romantic partner may also have lower levels of social support. Low levels of social support have been associated with poor mental health among women living with HIV [65, 66].

This study has limitations worth noting. All data were cross-sectional. The directionality of the relationship between IPV and mental health cannot be determined. Data were collected from pregnant women living with HIV in Yaoundé, Cameroon and may not be representative of pregnant women in other regions of Cameroon. Likewise, this study only represents women living with HIV who initiated PMTCT. We are missing those pregnant women living with HIV who either do not know their HIV status and/or who did not attend follow-up PMTCT services. If anything, this may result in an underestimate of CMD and IPV as lack of access to or utilization of antenatal health services could reflect both poor mental health or IPV that prevents service use. In addition, all participants were in their third trimester of pregnancy. The prevalence of probable CMD may be meaningfully different in the first or second trimesters of pregnancy, particularly as a meta-analysis of the prevalence of antenatal depression in Ethiopia found the prevalence of depression to be highest in the third trimester [62]. Additionally, to the authors’ knowledge, the SRQ-20 has not been formally validated for use in Cameroon. However, the SRQ-20 was developed to be used in LMICs and has been used widely throughout sub-Saharan Africa. Finally, the IPV questions were from the DHS, which was designed to increase disclosure and had been previously been used in Cameroon. However, they may still have reflected measurement errors. The questions were not culturally tailored to reflect any uniquely Cameroonian expressions or actions that would fall within the domains of IPV, and research has shown that IPV tends to be under-reported. Thus, IPV reported in this study may be an under-estimate of the true prevalence in our study population.

## Conclusions

Pregnant women living with HIV in Cameroon endorsed high prevalence of IPV and probable CMD. Screening and services to address IPV and mental health are urgently needed for this population. Interventions to both prevent and screen and address IPV and common mental health disorders should be implemented and evaluated.

## Data Availability

The dataset analyzed during the current study is available from the corresponding author on reasonable request and with permission of Catholic Relief Services.

## Abbreviations

aOR: adjusted odds ratio
ART: Antiretroviral therapy
CI: Confidence interval
CMD: Common mental disorders
DHS: Demographic and health survey
GEE: Generalized estimating eqs.
HIV: Human immunodeficiency virus
IPV: Intimate partner violence
LMIC: Low- and middle-income countries
mhGAP: Mental Health Gap Action Programme
PEPFAR: President’s Emergency Plan for AIDS Relief
PLWH: People living with HIV
PMTCT: Prevention of mother-to-child transmission of HIV
SES: Socioeconomic status
SRQ-20: WHO Self Reporting Questionnaire
UNAIDS: Joint United Nations Programme on HIV/AIDS
WHO: World Health Organization
